# New advances in perioperative cardioprotection

**DOI:** 10.12688/f1000research.17184.1

**Published:** 2019-04-24

**Authors:** Mona Momeni, Stefan De Hert

**Affiliations:** 1 Department of Anesthesiology & Acute Medicine, Cliniques universitaires Saint Luc, Université Catholique de Louvain, Institut de Recherche Expérimentale et Clinique, Pôle de Recherche Cardiovasculaire, Avenue Hippocrate, Brussels, 1200, Belgium; 2 Department of Anesthesiology & Perioperative Medicine, Ghent University Hospital, Ghent University, Corneel Heymanslaan 10, 9000 Ghent, Belgium

**Keywords:** Cardioprotection, Aging, Diabetes Mellitus

## Abstract

With the increasing age of the general population, medical conditions necessitating a surgical intervention will increase. Concomitant with advanced age, the prevalence of type 2 diabetes mellitus will also increase. These patients have a two- to three-fold higher risk of occurrence of cardiovascular events and are at higher risk of perioperative myocardial ischemia. This review will discuss recent advances in the field of perioperative cardioprotection and focus specifically on strategies that have aimed to protect the diabetic and the aged myocardium. This review will not deal with potential putative cardioprotective effects of opioids and anesthetic agents, as this is a very broad area that would necessitate a dedicated overview.

## Introduction

According to the US Census Bureau’s 2017 National Population Projections, there will be 78 million people 65 years or older in the US by 2035 ^1^. Elderly people are a growing part of surgical caseloads ^2^.

Also, with increasing age, the prevalence of ischemic heart disease increases. Ischemic heart disease is one of the leading causes of death worldwide ^3^. Similarly, the prevalence of type 2 diabetes mellitus increases in the proportion of people older than 65 years of age ^4^. Cardiovascular disease, especially ischemic heart disease, is an important risk factor of morbidity and mortality in patients with diabetes ^5^.

In this review, we outline recent cardioprotective strategies in patients with diabetes and in the elderly and discuss their eventual application in the perioperative setting. For a complete overview of the cardioprotective effects of routinely used anesthetics and other pharmacological agents commonly used in the perioperative period, we encourage the reader to address other systematic and narrative reviews in this field ^6– 14^.

## Type 2 diabetes mellitus

### Ischemia-reperfusion injury and cardioprotection in diabetes

Reperfusion of the ischemic myocardium is the main key to saving tissue. Nevertheless, reperfusion may result in harmful effects, known as reperfusion injury. The main mechanisms involved in the pathogenesis of reperfusion injury are calcium overload and oxidative stress with the production of reactive oxygen species. Other mechanisms are mitochondrial dysfunction, inflammation, apoptosis, endoplasmic reticulum stress, and protein kinase activation ^15, 16^.

Patients with diabetes seem especially vulnerable to the effects of myocardial ischemia-reperfusion injury. The exact underlying mechanisms are not fully known but an increased basal oxidative stress due to excessive reactive oxygen species production or reduced endogenous antioxidant defense system or both seem to play an important role ^17^. The enhanced basal oxidative stress is thought to be the result of chronic hyperglycemia. Chronic hyperglycemia as such severely impacts the ischemic myocardium. It is associated with endothelial dysfunction, impairs the development of coronary collateral blood flow, and attenuates the dilatation of coronary microcirculation in response to ischemia and to increased myocardial oxygen consumption ^18^. In addition, animal and human data support the concept that ischemic and anesthetic cardioprotective strategies are not effective in diabetic hearts ^19– 23^. Hyperglycemia further impairs the pharmacological activation of mitochondrial ATP-dependent potassium (K _ATP_) channels, responsible for preconditioning effects ^24^. Moreover, many patients with diabetes take sulfonylurea hypoglycemic agents which close the K _ATP_ channels. As activation of these channels is one of the mechanisms that protect the myocardium against ischemia-reperfusion injury, its blocking may explain the impaired cardioprotective effects of preconditioning strategies observed in many patients with diabetes.

### Metformin and its cardioprotective actions

Metformin is an oral anti-diabetic drug that is widely used in patients with diabetes. Its glucose-lowering effects result from various actions: inhibition of complex I of the mitochondrial respiratory chain, decreased hepatic glucose production, increased glucose reuptake, and stimulation of adenosine monophosphate-activated protein kinase (AMPK) ^25^. The main concern with the use of metformin in the perioperative period has been the development of lactic acidosis.

Interestingly, recent data show evidence for cardioprotective effects of metformin ^26^. The main action of metformin seems to be via activation of AMPK, which increases tolerance against ischemia-reperfusion injury. Activation of AMPK further results in phosphorylation of endothelial nitric oxide synthase (eNOS), increasing nitric oxide (NO) bioavailability and preventing mitochondrial permeability transition pore (mPTP) opening at reperfusion. Regulation of endothelial and myocardial NO synthesis by multi-site eNOS phosphorylation seems to be essential in the pathophysiology of different cardiovascular diseases and explains the beneficial effects on ischemia-reperfusion injury and heart failure ^27^. In addition, metformin stimulates intracellular formation of adenosine. Adenosine receptor stimulation activates the reperfusion injury salvage kinase (RISK) pathway, which in turn contributes to eNOS phosphorylation and prevents opening of mPTP ^28, 29^. Although beneficial effects of metformin on ischemia-reperfusion injury have been extensively shown in various small-animal models ^30– 35^, such effects could not be reproduced in swine models ^36^.

This is another example that data obtained on rodents should be confirmed by similar findings in larger-animal models before translating them into human research ^37– 39^.

They, moreover, highlight the influence of anesthetic agents when used in cardioprotective research. Indeed, in the study by Techiryan *et al*. ^36^, the pigs were maintained anesthetized with a continuous infusion of propofol. In a rat model, sevoflurane in the presence of low sedative propofol concentrations completely lost its protection ^40^. Otherwise, propofol has been shown to interfere with the cardioprotective mechanisms induced by remote ischemic preconditioning (RIPC) ^41, 42^. It is therefore plausible that propofol also interferes with other forms of cardioprotection.

### Metformin and clinical outcome data

Given its theoretical cardioprotective effects, metformin has been extensively studied in recent years. Many prospective and retrospective studies have shown the efficacy of metformin in decreasing cardiovascular events, mortality, and hospital readmission rates in patients with heart failure and diabetes ^43– 49^ and in patients with coronary artery disease and diabetes ^50– 54^. In non-diabetic patients, however, metformin seemed not to be associated with any beneficial effects in terms of cardiovascular outcome ^55– 59^. Whether in clinical practice the cardioprotective properties of metformin are more pronounced in diabetic hearts should be further investigated.

Otherwise, the positive effects of metformin were mostly obvious when it was used as chronic treatment. In trials where metformin was solely started before the initiation of the study, no beneficial effects could be observed.

In clinical practice, chronic metformin therapy in diabetic patients presenting for surgery is stopped because of the fear of perioperative lactic acidosis. In the era of perioperative cardioprotection, it is questionable whether this is justified. So far, no studies have investigated this issue.

### Newer anti-hyperglycemic medications and cardiovascular outcome data

Sodium-glucose cotransporter-2 inhibitors and glucagon-like peptide-1 receptor agonists are two new classes of anti-hyperglycemic agents ^60, 61^. Sodium-glucose cotransporter-2 inhibitors function by increasing urinary excretion of glucose in the renal tubules. Glucagon-like peptide-1 receptor agonists execute their function on the basis of the incretin effect, a response to release more insulin because of high glucose levels after a meal. The cardiovascular safety of both agents has been extensively evaluated in recent years. Although the results of randomized controlled trials with these new agents show cardiovascular safety, their beneficial effects in terms of cardiovascular outcome warrant further investigation ^62, 63^.

### Remote ischemic preconditioning and diabetes

RIPC is a technique during which brief periods of ischemia in a remote vascular bed provide protection against ischemia-reperfusion injury in different parenchymal organs. The most studied organ so far is the heart. It is beyond the scope of this review to discuss the putative mechanisms involved and the conflicting results of clinical trials on RIPC. The interested reader is referred to different review articles on the topic ^9, 64– 67^. The CONDI 2/ERIC-PPCI trial (ClinicalTrials.gov Identifier: NCT02342522), a large European multicenter study, is investigating whether remote ischemic conditioning prior to percutaneous coronary intervention in patients with ST-segment elevation myocardial infarction will decrease the rates of cardiac mortality and hospitalization for heart failure at 12 months.

Whether RIPC can have any positive influence on the diabetic myocardium has been evaluated in only a few trials. One of the studies that have specifically addressed this issue is a retrospective analysis ^68^ which showed that RIPC has no cardioprotective effects in patients with diabetes and may even be deleterious in those diabetics who received sulfonylurea hypoglycemic agents.

Recently, the EUROpean and Chinese Cardiac and Renal Remote Ischemic Preconditioning Study (EURO-CRIPS) has sought to determine whether RIPC could be cardioprotective in the presence of diabetes ^69^. Among 223 patients who underwent a percutaneous coronary intervention, 38% had diabetes mellitus. Periprocedural myocardial infarction occurred in a significantly higher number of patients with diabetes in the control group compared with the RIPC group.

The Effect of Remote Ischaemic Preconditioning on Clinical Outcomes in CABG Surgery (ERICCA) study ^70^ and the Remote Ischemic Preconditioning for Heart Surgery (RIPHeart) study ^71^ have looked at this issue as well. In both studies, the incidence of primary endpoint (death from cardiovascular causes, non-fatal myocardial infarction, coronary revascularization, or stroke for the ERICCA study and composite of death, myocardial infarction, stroke, or acute renal failure for the RIPHeart study) was similar between diabetic patients assigned to the control group and those assigned to the RIPC group.

Nevertheless, the use of propofol—known to interfere with the cardioprotective effects of remote ischemic conditioning—in both studies might have influenced their results ^72^. Therefore, further research and well-designed clinical trials are needed to seek whether diabetic myocardium is responsive to the cardioprotective effects of RIPC.

### Beta-blockers and their cardioprotective effects in diabetes

Beta-blockers are recommended as cardioprotective medication in patients with coronary artery disease ^73^ and congestive heart failure ^74^. Indeed, their use is associated with reduced mortality and reduced recurrent myocardial infarction after myocardial infarction. Beta-blockers decrease mortality as well in patients with chronic heart failure and systolic dysfunction. Patients with diabetes often present different cardiovascular risk factors. The Diabetes Postoperative Mortality and Morbidity (DIPOM) trial evaluated the long-term effects of 100 mg metoprolol or placebo on mortality and morbidity in diabetic patients undergoing major non-cardiac surgery ^75^. This study was unable to show the benefit of starting β-blockers in the perioperative setting in patients with diabetes. Very recently, the relationship between the use of β-blockers and all-cause mortality was evaluated in patients with diabetes mellitus and those without ^76^. The mortality of diabetic patients taking β-blockers was higher compared with those diabetics who did not take β-blockers (hazard ratio 1.65, 95% confidence interval 1.13–2.40; *P* = 0.009). Similar results were found when only β1-selective β-blockers were taken into analysis. However, all-cause mortality was significantly lower in non-diabetic patients taking β1-selective β-blockers compared with non-diabetic participants not taking β-blockers ( *P* = 0.01). Although the authors cannot explain the exact reason for these observed differences, they hypothesize that adverse effects on glucose metabolism (more hypoglycemia and hypoglycemia unawareness in diabetics) and weight gain induced by β-blockers may result in an increased risk of mortality.

What is true from a cardioprotective perspective in non-diabetics may not necessarily be relevant in patients with diabetes mellitus. Further research in this field is mandatory before drawing any firm conclusions.

## Aging

### Ischemia-reperfusion injury and cardioprotection in the aged myocardium

Aging induces structural and functional changes in the heart, as in all other human organs, resulting in greater damage of the aging heart owing to the deleterious effects of ischemia-reperfusion injury ^77– 79^. Moreover, experimental studies have shown that the aging myocardium is less responsive to ischemic preconditioning ^80– 84^. This reduced preconditioning effect in the aged myocardium has also been observed with inhalational anesthetics ^85, 86^.

A recent study specifically investigated the influence of aging on the release of cardioprotective humoral factors after RIPC and the cardioprotective effects of RIPC on aged myocardium ^87^. From the data obtained in this study, it appears that the release of humoral factors after RIPC is age-dependent and that the RIPC-induced humoral factors are cardioprotective also in the aged heart. These results emphasize the complex mechanisms involved in the cardioprotective effects of RIPC and might partly explain the disappointing observation in large clinical trials aiming to show the perioperative cardioprotective effects of RIPC ^70, 71^.

### Studies of perioperative cardioprotection taking into account patient’s age

Despite the existing evidence of experimental trials that the aged myocardium is less responsive to any type of cardioprotection, few clinical studies have clearly analyzed the possible relation between the extent of perioperative cardioprotection and age.

From the available clinical data, it is not clear whether considerable cardioprotection can be achieved in the elderly ^70, 88– 91^. Of note, it remains difficult to give an exact definition of “old myocardium”. More than the chronological age of the patient, conditions that influence the endogenous protective mechanisms of the myocardium might affect the response of the heart to various protective mechanisms. Physical activity has been shown to be among such protective mechanisms ^92, 93^. Future trials need to take into account these aspects.

### Comedication

Elderly people often take various cardiovascular (and other) medications. These treatments alone or in combination may interfere with cardioprotective mechanisms. Some of these drug therapies have been discussed in this review article. Other routinely used medications that have been studied in the context of perioperative cardioprotection and that will be further discussed in this review article are (1) statins, (2) angiotensin-converting enzyme inhibitors (ACE-Is)/angiotensin receptor blockers, (3) calcium channel blockers, and (4) nitrates. The interaction of these drugs with some of the perioperative cardioprotective strategies has been studied in different trials.

***Statins.*** In recent years, much interest has been given to the pleiotropic effects of statins, contributing to their cardioprotective effects ^94^. The cardioprotective properties of statins have been evaluated in numerous trials resulting in a considerable number of meta-analyses and systematic reviews. It seems that perioperative statin therapy is associated with a lower incidence of postoperative myocardial infarction in non-cardiac surgery but not in cardiac surgery. The pathophysiology of perioperative myocardial ischemia is different in non-cardiac ^95^ and cardiac surgery, which may explain the discrepant results between the two surgical groups.

Based on the evidence available in 2014, the European Society of Cardiology/European Society of Anaesthesiology (ESC/ESA) guidelines on non-cardiac surgery have a class I recommendation for perioperative continuation of statins, favoring statins with a long half-life or extended-release formulation. A class IIa recommendation has been given for preoperative initiation of statin therapy in patients undergoing vascular surgery and ideally this should be performed at least 2 weeks before surgery ^96^.

***Angiotensin-converting enzyme inhibitors and angiotensin receptor blockers.*** A large meta-analysis of randomized clinical trials of renin–angiotensin–aldosterone system inhibitors in patients with hypertension showed that all-cause mortality was significantly reduced with these drugs compared with controls ^97^. However, the observed treatment effect resulted from the ACE-Is. This decrease in mortality could not be demonstrated with angiotensin receptor blockers ^97^.

Both drugs, when used in the perioperative period, can induce mild to severe hypotension, which can be resistant to vasopressors in some patients. Therefore, when these drugs are used for hypertension, their withdrawal 24 hours before surgery has been recommended. This is a class IIa recommendation from ESC/ESA guidelines on non-cardiac surgery ^96^. These guidelines recommend continuation of ACE-Is or angiotensin receptor blockers under close monitoring during non-cardiac surgery in stable patients with heart failure and left ventricular systolic dysfunction (IIa).

***Calcium channel blockers.*** Few well-powered studies have evaluated the beneficial effects of calcium channel blockers in the perioperative period. The safety and efficacy of these drugs have been questioned ^98^.

The use of dihydropiridine calcium channel blockers has been associated with 30-day mortality in patients with acute or elective aortic aneurysm surgery ^99^. In this regard, the 2014 ESC/ESA guidelines recommend that the continuation or introduction of heart rate–reducing calcium channel blockers may be considered in patients not tolerating β-blockers ^96^.

***Nitrates.*** In recent years, there has been increasing interest in the cardioprotective effects of nitrates. Previous studies have shown that intravenous injection of nicorandil can decrease the incidence of myocardial injury after percutaneous coronary intervention ^100, 101^. Otherwise, a single oral dose of nicorandil showed cardioprotective effects after coronary angioplasty ^102^. The preconditioning actions of nitroglycerin have been demonstrated in specific clinical scenarios ^103, 104^. Leesar *et al*. ^103^ showed, for the first time, that 4-hour intravenous administration of nitroglycerin protected human myocardium against ischemia 24 hours after its administration ^105^. Nevertheless, the endothelial preconditioning effects of a single dose of nitroglycerin are lost after a prolonged exposure to nitroglycerin, limiting the beneficial effects of long-term prophylactic nitrate therapy ^106^. In a small study, its acute administration did not interfere with remote ischemic conditioning ^107^. Otherwise, two large clinical studies failed to show any possible benefit in terms of mortality with the use of nitrates in the setting of acute myocardial injury with thrombolytic therapy ^108, 109^.

## Conclusions

Prevention of perioperative morbidity and mortality is a challenge in patients with diabetes and in the elderly. Patients with diabetes are at high risk of ischemic heart disease and are vulnerable to the effects of myocardial ischemia-reperfusion injury. Chronic metformin treatment has shown promising results regarding cardiovascular outcome in patients with diabetes. Its perioperative cardioprotective effects still need to be investigated. Nevertheless, its withdrawal before any surgery may not be justified, as the risk of lactic acidosis is extremely low. The safety and efficacy of RIPC in the setting of diabetes need to be elucidated. Otherwise, β-blockers may not have the same beneficial effects in diabetic patients compared with non-diabetics. Their use should be carefully evaluated in diabetic patients who have a maximum medical treatment.

Advanced age is often associated with cardiovascular morbidity. Currently, there is no clear evidence whether elderly patients are less responsive to routine perioperative cardioprotective strategies. Comedication is often observed in older patients. Current evidence strongly supports the continuation of statins in the perioperative period. ACE-Is reduce all-cause mortality when used for hypertension and should only be stopped 24 hours before surgery to avoid hypotension.

In conclusion, the translation of cardioprotection into the clinical setting where advanced age and various comorbidities are common calls for well-designed experimental and clinical studies.

## Abbreviations

ACE-I, angiotensin-converting enzyme inhibitor; AMPK, adenosine monophosphate-activated protein kinase; eNOS, endothelial nitric oxide synthase; ERICCA, Effect of Remote Ischaemic Preconditioning on Clinical Outcomes in CABG Surgery; ESA, European Society of Anaesthesiology; ESC, European Society of Cardiology; K _ATP_, ATP-dependent potassium; mPTP, mitochondrial permeability transition pore; NO, nitric oxide; RIPC, remote ischemic preconditioning
